# Processing of Scene-Grammar Inconsistencies in Children with Developmental Language Disorder—Insights from Implicit and Explicit Measures

**DOI:** 10.3390/brainsci15020139

**Published:** 2025-01-30

**Authors:** Daniela Bahn, Dilara Deniz Türk, Nikol Tsenkova, Gudrun Schwarzer, Melissa Le-Hoa Võ, Christina Kauschke

**Affiliations:** 1 Clinical Linguistics, German Linguistics, University of Marburg, 35037 Marburg, Germany; kauschke@uni-marburg.de; 2 Scene Grammar Lab, Department of Psychology, Goethe University Frankfurt am Main, 60323 Frankfurt am Main, Germany; d.tuerk@psych.uni-frankfurt.de (D.D.T.); mlvo@psych.uni-frankfurt.de (M.L.-H.V.); 3 Developmental Psychology, Psychology and Sport Science, Justus Liebig University Giessen, 35394 Giessen, Germany; nikol.tsenkova@psychol.uni-giessen.de (N.T.); gudrun.schwarzer@psychol.uni-giessen.de (G.S.); 4 Neuro-Cognitive Psychology, Department of Psychology, Ludwig-Maximilians-University, 80539 Munich, Germany

**Keywords:** DLD, language skills, visuospatial cognition, scene grammar, eye tracking, semantic and syntactic violation

## Abstract

Background/Objectives: Developmental language disorders (DLD) are often associated with co-occurring neurodevelopmental difficulties, including attentional or social–emotional problems. Another nonverbal domain, i.e., visual cognition and its relationship to DLD, is virtually unexplored. However, learning visuospatial regularities—a scene-grammar—is crucial for navigating our daily environment. These regularities show certain similarities to the structure of language and there is preliminary evidence for a relationship between scene processing and language competence in preschoolers with and without DLD. This study compared implicit and explicit visuospatial knowledge of everyday indoor scenes in older children, aged 6 to 10 years, of both groups. Methods: We measured ‘dwell times’ on semantic and syntactic object—scene inconsistencies via eye-tracking and performance in an object-placement task, and their associations with children’s language, visual, and cognitive skills. Results: Visual attention towards object-scene inconsistencies was highly comparable between groups, but children with DLD scored lower in a visual perception test and higher language skills were associated with higher visuo-cognitive performance in both tasks. In the explicit scene-grammar measurement, this relationship only existed for children with DLD and disappeared when nonverbal cognitive performance was controlled. Conclusions: Our study suggests the existence of mild problems in visuospatial processing co-occurring with DLD, which is partly influenced by age and nonverbal cognitive ability. The acquisition of visual cognition and linguistic knowledge is an interactive, multimodal process where the perception of objects in scenes might affect how the words for these objects are learned and vice versa. A better understanding of this interplay could eventually have impact on the diagnosis and treatment of DLD.

## 1. Introduction

From their very first day, babies begin to visually explore their surroundings. By accompanying their caregivers everywhere, they have the opportunity to observe their everyday environment constantly and repeatedly. This environment is not static, but often changes in front of the baby’s watchful eyes. Imagine that a child observes their mother standing in the kitchen and preparing lunch, while she grabs, manipulates, and replaces different objects—just one of many complex visual scenes the child is confronted with every day. By comparing this visual spatial input with previously gathered information relating to the same or similar scenes (e.g., other kitchens), the child extracts important visuospatial regularities [1]. With this implicitly acquired ‘set of rules’, children learn to efficiently understand scenes, recognize the objects embedded within them, and guide goal-directed behavior and, finally, can predict which objects usually appear where within a scene [1]. This enables them to navigate even completely unfamiliar surroundings. For example, our previous experience with bathrooms will generate an expectation of finding the soap on the sink, and not next to the toilet, in every new bathroom we will enter in life. Thus, learning about objects and their locations, i.e., the acquisition of scene knowledge, is a crucial developmental challenge.

### 1.1. Scene-Grammar Structure

According to Võ [1], visual scenes are governed by rules underlying the placement of objects that are part of the scenes. Given some structural similarities between scenes and language, the term “scene grammar” has been coined. In language, words serve as the basic units, each carrying specific semantic meaning. Words combine to form larger units such as phrases (groups of words) and sentences (ordered combinations of phrases following syntactic regularities). Sentences are composed of obligatory constituents—such as the predicate and its arguments (e.g., ‘Peter*_subject_* is laughing*_predicate_*’)—as well as optional constituents (adjuncts) that “provide all sorts of additional information about the event/state” [2] (p. 259) (e.g., ‘Peter*_subject_* is laughing*_predicate_* in the kitchen*_adverbial phrase_*). In a similar way, a scene (e.g., a kitchen) is built upon several meaningful subgroups. Võ [1] uses the linguistic term ‘phrases’ to refer to these subgroups of objects, drawing a parallel to phrases in language. Each phrase contains an obligatory, global object—referred to as an ‘anchor’– which is large, static, and often diagnostic for the scene context (e.g., the oven in a kitchen). Anchors are accompanied by one or more smaller optional elements, which are called local objects (e.g., a pot on the oven). In terms of space and position, anchor objects and local objects are closely related in that anchors predict the location and identity of local objects [1]. Thus, language and scenes share their hierarchical structure, the presence of both obligatory and optional elements, and rules for combining these elements.

In experimental settings, the structure of sentences and scenes can be manipulated in terms of form and/or content. Semantic violations in linguistic stimuli occur when the meaning of a word is incongruent with the context of the sentence (e.g., ‘Peter swims in the kitchen’), while syntactic errors arise when the word order in the sentence is violated (e.g., ‘Peter in the kitchen laughing’). Such violations usually lead to higher cognitive costs in language processing [3]. In a metaphorical way, visuospatial scenes can also be either semantically or syntactically inconsistent. Semantic violations occur when an object does not fit the scene, such as a bike helmet in the oven instead of a cake. Syntactic violations, on the other hand, involve typical objects positioned in atypical locations (e.g., a cake located in the dishwasher and not in the oven). Identifying such inconsistencies within a scene is associated with longer gaze durations, reflecting extended processing demands, as demonstrated in several eye-tracking studies [4,5,6,7]. These findings highlight parallels between the processing of language and the processing of scenes.

Given the multimodal nature of the sensory input in our daily lives, processing of scenes and language takes place in parallel. While children observe certain visuospatial scenes, they are simultaneously exposed to linguistic input [8]. They hear words for certain objects that are present in a scene. Thus, linguistic input is important to acquire mental representations for different components of visuospatial scenes [7]. Besides this obvious link, there might be a deeper connection relative to the development and processing of scene grammar and language, grounded in common underlying cognitive processes as suggested by a positive relationship between linguistic skills and the efficiency of scene-grammar processing [7]. Thus, children who experience difficulties in organizing and interpreting linguistic structures may also face challenges in constructing coherent scene representations. However, research into the development of scene-grammar processing is still in its early stages. Building upon this, our study aimed at uncovering whether and how language competence drives the visuospatial processing of indoor scenes. Assuming a link between the visual and language domains, a comparison of scene-grammar processing in children showing various levels of language competence might be the ideal test case. In this study, we therefore explored scene-grammar knowledge in a group of children with typical language skills and a group of children with developmental language disorder (DLD); the latter is characterized by difficulties in linguistic domains such as lexicon, semantics, or grammar.

### 1.2. Developmental Language Disorder

Childhood language disorders are deviations in children’s typical language acquisition that, without being treated, may lead to serious negative consequences for social interactions and participation, wellbeing, and educational and career success. With a prevalence of approx. 10%, they are one of the most common developmental disorders in childhood [9,10]. For the majority of children, their language disorder is of unknown origin, i.e., not associated with a known differentiating condition (e.g., a genetic syndrome) with deficits appearing mainly in the language domain. This sort of language impairment has traditionally been referred to as specific language impairment (SLI). However, the invoked specificity has been questioned by empirical evidence as well as clinical experience demonstrating that affected children often show additional delays, impairments, or underachievement in other developmental areas [10,11,12]. This debate finally led to several multinational and multidisciplinary consensus studies, initiated in English-speaking countries by the CATALISE consortium [10,11], which aimed at a comprehensive revision of SES terminology and diagnostic criteria, based on consecutive expert surveys in the relevant disciplines. In German-speaking countries, the D-A-CH Consortium-SES pursued this purpose [12]. As a result of these efforts, the interdisciplinary use of the term DLD, instead of SLI, has been established for language disorders with no known differentiating condition.

A high degree of consensus has also been reached that children with DLD show heterogeneous profiles of individual strengths and weaknesses with persistent receptive and/or expressive deficits in at least one linguistic domain (i.e., phonology, lexicon and semantics, morphology and syntax, pragmatics, and social communication [10,11,12]). Phonological problems [13] affect a child’s ability to select and combine speech sounds to form correct sound sequences and word forms. Phonological errors such as substitution or omission of speech sounds can reduce a child’s intelligibility. Lexical-semantic disorders [14] manifest themselves in a restricted vocabulary, poor understanding of word meaning, or problems in retrieving words from the lexicon (word-finding difficulties). Grammatical impairment [15] is characterized by difficulty in understanding and using the morphological and syntactic rules of a given language. Symptoms of grammatical impairment are varied and depend on the structure of the target language [16]; they include errors in morphological marking, omission of obligatory constituents or function words (e.g., articles, prepositions), and word-order errors. Finally, social pragmatic difficulties [17] may impede the appropriate use of verbal and nonverbal communicative means in a given situation, the processing of figurative language, or narrative and conversational skills. In the context of the present study, grammatical and lexical-semantic symptoms of DLD are particularly relevant because they are most likely to be related to the processing of scene grammar.

A key element of the new definition is the notion of co-occurring conditions, i.e., “impairments in cognitive, sensorimotor or behavioral domains that can co-occur with DLD and may affect pattern of impairment and response to intervention, but whose causal relation to language problems is unclear. These include attentional problems (ADHD), motor problems (developmental coordination disorder or DCD), reading and spelling problems (developmental dyslexia), speech problems, limitations of adaptive behavior and/or behavioral, and emotional disorders”. [11] (p. 1072). Consequently, a low level of cognitive ability no longer precludes a diagnosis of DLD [10]. The existence of such co-occurring conditions supports a domain-general nature of DLD. However, it is an open question as to what kind of neurodevelopmental problems may accompany the language disorder. In particular, it is unclear whether visual cognition is also a vulnerable domain in DLD. Initial empirical evidence points in this direction, but is inconsistent, depending on the experimental tasks and the memory systems activated [18].

### 1.3. Visuospatial Processing in DLD

Visuospatial ability refers to a person’s capacity to identify, integrate, and analyze visual information relating to patterns and objects and to understand their spatial relationships. It covers attentional processes that mediate the selection of relevant and the suppression of irrelevant visual information as well as the activation of memory systems involved in the perception, manipulation, and imagination of objects in space—both in static and in dynamic displays as well as across several dimensions [19]. Short term memory (STM) allows keeping information activated for a short amount of time and is usually measured via span tasks, in which participants listen to an order of words or digits, trying to recall as many as possible. Conceptually similar, but with minimal verbal mediation, ‘corsi block tasks’ assess visual STM by having participants recall sequences of squares on a 9 × 9 block layout that had been highlighted in color. In contrast to STM, working memory (WM) is involved in more complex cognitive processes that require not only recall, but also the flexible manipulation of information such as learning and language processing. WM is often measured via dual tasks, span tasks or corsi block tasks with backward recall. According to Baddeley’s model of working memory [20], it consists of a central executive that controls two slave systems as well as an episodic buffer which integrates the information of both systems: the phonological loop for maintaining and processing verbal information, often impaired in children with DLD, and the visuospatial sketchpad for keeping information about spatial relationships and object properties for further processing. Some studies reported poorer performance of children with DLD in verbal span tasks, but equal performance in nonverbal visuospatial STM and WM tasks [21,22,23,24,25], and interpreted this pattern in favor of a domain-specific view as to DLD. In contrast, there is growing evidence that children with DLD have deficits in both verbal and visual attention [18,26,27,28], supporting the domain-general processing limitation account [29]. Arslan and colleagues [18] investigated nonverbal and verbal STM and WM with span and corsi block tasks in children and adolescents with DLD (aged 7–11 years and 12–18 years) and found visual and verbal components to be modulated differently by the factor of age. While both groups with DLD showed poorer performance compared to TD controls in the verbal domain, visual STM and WM were only impaired in the younger group with DLD. A meta-analysis [30] on the effect sizes of 21 studies comparing the performance of children with DLD and TD peers in visuospatial WM tasks (involving central executive and storage) showed that higher deficits in visuospatial processing were associated with a more pervasive language impairment. In addition, Gray and colleagues [31] investigated the performance of 300 children with DLD, dyslexia, or both in 13 tasks which assessed central executive, phonological, and visuospatial/attention components of WM. For 21% of the children with DLD they reported poorer performance in all WM components compared to the TD controls.

The third memory system, the visual long-term memory, stores conceptual knowledge about object properties and their spatial relations. It is commonly divided in declarative (episodic and general semantic) knowledge and procedural memory. Nonverbal (including visual) declarative knowledge, tested by memory tasks using pictures or abstract patterns, appears to be relatively intact in DLD [32]. In terms of procedural memory, children with DLD often respond slower not only in verbal tasks (e.g., naming), but also in nonverbal tasks, such as categorization [33] or interference control (e.g., ‘stroop tasks’), as recently shown in a meta-analysis comprising 46 studies on children with DLD [34].

As mentioned earlier, visual and verbal processing often run in parallel, and sometimes conclusions about procedural efficiency in one modality can be drawn from observing behavioral responses in the other. Lara-Diaz and colleagues [35] measured phonological processing via visual attention in an auditory visual-world identification task combined with eye-tracking in children diagnosed with DLD. The children heard a word and were asked to look at the target picture, which was displayed next to pictures with phonologically related distractors (rhyme and the same onset) and one unrelated distractor. Compared to their TD peers, children with DLD showed a reduced sensitivity towards the identification of rhyme words, as seen in the lack of an interference effect (longer looking times relative to phonological distractors compared to unrelated objects while searching for the target). Visual scenes, however, are more complex than single objects (comparable to the word-sentence distinction in language). Therefore, methods using the visual-world paradigm and eye-tracking seem to be a more worthwhile and ecologically valid way to investigate children’s knowledge of the structure of everyday visual scenes. Such investigations may also shed light on the role of language competence in efficient scene-grammar processing. To date, only a few studies have used this combination of methods in comparing children with typical language development and those with DLD to investigate the acquisition of scene-grammar regularities.

### 1.4. Accessing Scene Grammar in Children with and Without DLD Using Eye-Tracking

Previous studies with TD children at a preschool age investigated how scene-grammar knowledge guides children’s visual attention during free observation of pictures displaying everyday indoor scenes, e.g., bathrooms or bedrooms [7,36,37]. These scenes were either consistent (scene-related objects appear at expected locations), semantically inconsistent (objects do not fit the scene context), syntactically inconsistent (objects appear at unexpected locations), or both semantically and syntactically inconsistent. The results provided the first evidence that already by the age of 2, children are able to perceive semantic and syntactic violations, as indicated by inconsistency effects (longer observation of inconsistent, compared to consistent, objects) [7,36] and that inconsistency effects for combined semantic and syntactic violations and for pure semantic violations appear earlier in the course of development than those for pure syntactic violations. With increasing age, inconsistency effects become stronger with gaze durations on consistent objects decreasing, in relation to inconsistent objects [7]. In contrast to adults, inconsistency effects in children are driven by saliency, with stronger effects for violations with high visual saliency [36]. The existence of inconsistency effects in early childhood has been further supported by an ERP study which found that children at the age of two already showed adult-like ‘N400 effects’ when observing semantic scene inconsistencies, supporting the assumption that they are able to detect them implicitly [37]. In addition to the free-viewing paradigm combined with eye-tracking, Öhlschläger and Võ [7] directly accessed scene-grammar knowledge of preschool children (2–4 years), using a behavioral task in which children were asked to furnish a wooden dollhouse. The house included four rooms, specifically, a bedroom, kitchen, living room, and bathroom that were predefined by the anchor objects bed, oven, sofa, and shower. The authors found an age-related increase relative to correctly placed objects and closer inter-object-relations, indicating knowledge regarding the spatial structure of anchors and local objects in indoor scenes. They also found correlations between implicit and explicit measures of scene knowledge: a reduction in first-pass dwell times on consistent objects (strength of inconsistency effect) was predicted by the object placement performance in the dollhouse task. Furthermore, the aforementioned studies report positive correlations between congruency effects and expressive vocabulary, indicating that a high level of language skills is associated with a higher sensitivity towards scene violations (i.e., stronger inconsistency effects) [7,36] and a higher object placement performance in the dollhouse task [7].

As mentioned above, children with DLD often have problems with the acquisition of grammatical structures, as evidenced by a reduced ability to detect syntactic errors [38]. The acquisition and correct use of grammatical rules requires procedural memory capacity. The domain-general processing limitation account [29] and the procedural deficit hypothesis (PDH) [39,40,41] view DLD as a complex neuropsychological condition and suggest that the language processing deficits seen in DLD may be due to impaired statistical learning, i.e., an underperformance of domain-general mechanisms involving the central executive, STM and WM. According to the PDH, this underperformance results from abnormalities in brain structures that mainly affect the basal ganglia—a structure responsible for higher cognitive functions such as perception, attention, and executive functions, and thus for the constitution of procedural memory. Deficiencies in procedural memory may therefore affect language and visuo-cognitive processing in the same way. For example, possible delays in the acquisition of visual scene knowledge may affect the acquisition of words for spatial relationships, such as prepositions. In view of this, children with DLD may also have difficulty recognizing semantic and syntactic inconsistencies in scene grammar. Investigating visuo-cognitive performance, including the processing of everyday visual scenes, in children with DLD may provide further evidence for the domain-general explanation of DLD.

To our knowledge, scene-grammar processing in children with DLD has only been tested in one study. Applying the same experimental procedure as with TD children [36], Helo et al. [42] investigated whether 5;4- to 6;6-year-old children with DLD differ from TD peers as to gaze behavior when observing pictures showing consistent or semantically or syntactically inconsistent indoor scenes. The authors found group differences regarding the emergence of consistency effects. Children with DLD showed inconsistency effects only for the very obvious semantic and syntactic violations, but not for the syntactic-only and semantic-only conditions. Thus, children with DLD were less attracted by scene inconsistencies compared to TD peers, which speaks for an existing link between language and scene-grammar processing.

However, no direct group comparisons of the mentioned eye tracking measures were reported in this study, and the results were based on only one age-group of preschool-children and one task. It remains unclear whether a reduced sensitivity to syntactic scene violations might depend on age. Older children of primary-school age may still show different gaze behavior towards scene inconsistencies, or they may have caught up with their TD peers. Furthermore, the use of more than one experimental task and the inclusion of multiple measures of lexical and grammatical ability will shed light on the extent to which scene-grammar processing is less efficient in children with DLD, and how strongly it is related to language ability.

### 1.5. Objective

Against this background, the aim of the present study was to assess the scene-grammar processing of TD children and children with DLD of primary-school age in two different ways: (1) implicitly, by analyzing children’s looking behavior relating to congruent and inconsistent indoor scenes in a free-viewing eye-tracking paradigm, and (2) explicitly, by using a dollhouse task. We chose two nonverbal tasks that did not require any language processing other than understanding the instructions, as this minimized the likelihood that poorer performance in scene-grammar processing by the DLD group was due solely to their language deficits. Furthermore, we comprehensively tested the participants’ linguistic, nonverbal cognitive, and visual skills to uncover potential relationships relative to scene-grammar processing. Lower performance in scene-grammar processing, i.e. a reduced sensitivity towards semantic and syntactic scene violations, as well as a reduced ability to arrange objects in appropriate spatial relations and locations, would support the notion that visual and verbal processing may be grounded in the same underlying cognitive processes and, at the same time, contribute to a more comprehensive clinical picture of this multifactorial disorder.

## 2. Materials and Methods

### 2.1. Participants

The sample consisted of two groups of children aged from 5 years and 7 months up to 10 years and 10 months of life. The first group included 20 participants (4 female) with typical language development (TD) and the second group 20 children (4 female) with a diagnosis of DLD showing receptive and/or productive deficits in lexical/semantic and/or grammatical abilities. Thus, children with speech sound disorders only were not included in the sample. Children of both groups underwent subtests from two standardized developmental language tests to confirm the diagnosis of DLD and typical language development, respectively (see Section 2.2 and Section 3.1, including Table 1). Participants of both groups were matched in pairs according to gender and months of life (*M*_DLD_: 95.8 months, *SD*_DLD_: 22 months; *M*_TD_: 96.2 months, *SD*_TD_: 21 months). To examine potential age effects, we split both groups at the age of 7.88 years. This resulted in two age groups: one consisting of participants aged 5.58 to 7.92 years (*N*_TD_ = 9; *N*_DLD_ = 11) and the other aged 8.92 to 10.83 years (*N*_TD_ = 11; *N*_DLD_ = 9).

TD children were recruited via schools and local day care services, and by using public announcements. Parents of children with DLD were informed about the study by their children’s speech and language pathologists. All children had been receiving speech and language therapy for several months (at least once a week) through local services. With the exception of one child, all children were also attending intensive in-patient therapy at speech and language intervention centers at the time of testing. The parents were financially compensated, and the children received a small gift after each session.

### 2.2. Procedure

Prior to the experimental procedure, children and parents were fully informed about the purpose and content of the study. Parents agreed to their children’s participation and on the recording of audio and video clips of their children’s responses by signing informed-consent documents. Children verbally agreed to participate.

The study comprised three sessions, which took approximately 40 min each. They were conducted in a quiet room, either in laboratories at the Universities of Frankfurt am Main, Giessen, and Marburg (Hesse, Germany); at intervention centers for speech and language therapy; or at the participants’ homes. The first session included the implicit and explicit measure of scene-grammar processing, i.e., the free-viewing task (Eye-Tracking, see Section 2.3) and the dollhouse task (see Section 2.4).

In the second session, we assessed children’s nonverbal cognitive skills and visuospatial perception with three different tests to rule out any potential problems in scene-grammar processing in children with DLD which might be due to attention deficits or reduced intellectual abilities (see Section 3.2 and Table 1 for results). (1) In the *attention test—star search* (subtest 3 of the ‘Sprachstandserhebungstest für Kinder im Alter zwischen 5 und 10 Jahren—SET 5–10′ [43]), children were asked to identify as many stars as possible within lines of mixed symbols in one minute. (2) We also conducted the nonverbal intelligence test ‘Coloured Progressive Matrices—CPM’ [44]. (3) Children completed three subtests of the German adaption of the ‘Developmental Test of Visual Perception’—DTVP-3 [45], i.e., ‘Frostigs Entwicklungstest der visuellen Wahrnehmung—FEW-3′ [46]. The subtests were figure–ground (recognition of hidden forms against noisy backgrounds), visual closure (finishing forms mentally), and form constancy (recognizing identical shapes regardless of their size, color, or orientation).

Language testing took place in the third session (see Section 3.1 and Table 1 for results). We applied five subtests of the ‘SET 5–10′ [43]: expressive vocabulary (words and categories), sentence comprehension, text comprehension, error identification, or sentence correction (based on the participants’ age). As the inclusion criterion for the experimental group, children with DLD should either perform 1.5 SD below age-specific norms (percentile rank < 7) in at least one subtest of the SET 5–10 or between 1 and 1.5 SD below the norm (percentile rank 7–16) in at least three subtests. TD children were allowed, in only one of the subtests, a performance of 1 to 1.5 SD below the norm (percentile rank 7–16). Besides the group assignment of participants, language skills were also obtained to investigate their impact on experimental performance in implicit and explicit scene-grammar processing. We therefore conducted the subtest *picture description* of the ‘Patholinguistische Diagnostik bei Sprachentwicklungsstörungen—PDSS’ [47]. This instrument utilizes elicited production in order to generate four test scores: mean length of utterances, completeness of utterances, and two grammar scores comprising relevant target structures. The construction of the grammar scores was inspired by the Index of Productive Syntax (IPSyn), and modified for the German language [48].

### 2.3. Implicit Measure of Scene Knowledge: Free-Viewing Task

#### 2.3.1. Stimuli

**Figure 1:**
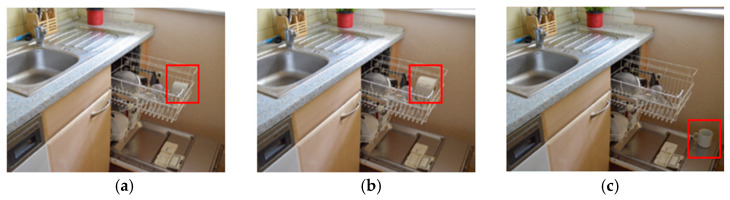
Examples of a subset of SCEGRAM scene pictures [4]: (**a**) mug in dishwasher = consistent; (**b**) toilet paper in dishwasher = semantic violation; and (**c**) mug at a wrong location in the dishwasher = syntactic violation.

A total of 45 images were selected from the SCEGRAM database [4], encompassing both semantically inconsistent and syntactically inconsistent scenes, as well as consistent scenes (see Figure 1 for an example). We created areas of interest (AOIs) which retained their size and location consistency across both the semantically inconsistent and consistent conditions. Similarly, in the syntactically inconsistent and consistent conditions, the AOIs maintained identical sizes. We employed less stringent eye-tracking thresholds for children by including an online buffer of 75 pixels on each side of the AOI. Images were counterbalanced across participants, ensuring that each participant encounters an object only in one condition. To rule out the potential influence of saliency on the differences between conditions, we computed the mean saliency rank using DeepGaze IIE [49]. Analysis revealed no difference between our conditions (consistent/semantically inconsistent—ratio = 0.822, SE = 0.139, z ratio = −1.16, *p* = 0.48; consistent/syntactically inconsistent—ratio = 0.795, SE = 0.134, z ratio = −1.364, *p* = 0.36; semantically inconsistent/syntactically inconsistent—ratio = 0.966, SE = 0.162, z ratio = −0.204, *p* = 0.98).

#### 2.3.2. Apparatus

We tracked children’s eye movements from the left eye, employing the EyeLink 1000 Portable Duo system (SR Research, Kanata, Ontario, Canada) with a sampling rate of 500 Hz, operated in remote mode. Stimuli were presented either on a 17-inch laptop screen or a 24-inch monitor with a resolution of 1920 × 1080 pixels and a refresh rate of 144 Hz. Children were seated approximately 70 cm away from the screen. Before starting the experiment, a 5-point calibration was performed using an audio–visual target and a drift check was performed every 10 trials. After 2 practice trials, children proceeded to the main experiment of 45 trials. Each trial started with an audio–visual fixation spiral, appearing to the left or right side of the screen. The fixation spiral was gaze-contingent, requiring children to maintain fixation for at least 500 milliseconds to trigger the stimulus presentation. The scene images appeared on the screen for 7 s, with a reward video randomly shown approximately every 2 images, lasting about 10 s each time. Children were instructed to freely observe the scenes (see [7] (p. 7) for an image of the trial sequence of this gaze-contingent eye-tracking paradigm).

#### 2.3.3. Analysis of Eye-Movements

To investigate children’s interest in scene violations, we focused on total dwell time (DT) as an eye movement measure. Dwell time is the sum of all fixations and saccades from the first to the last visit to a specific area of interest, reflecting the interest shown as to that area. We excluded trials with a dwell time of less than 100 ms (8.4%) from our analysis, as such brief durations are typically considered insufficient for processing information [50].

### 2.4. Explicit Measure of Scene Knowledge: Dollhouse Task

**Figure 2:**
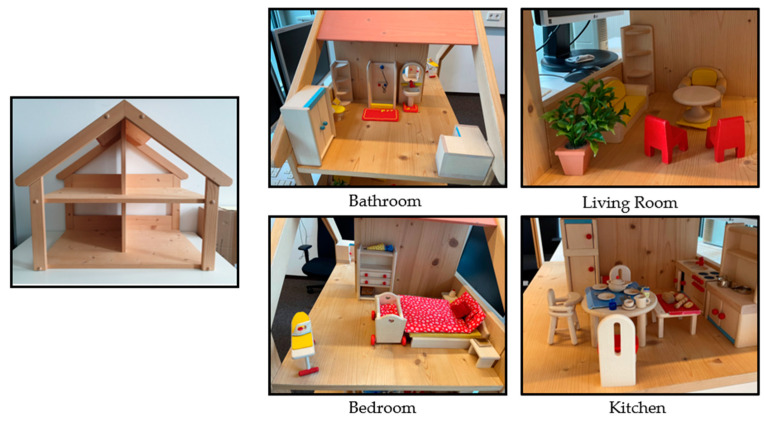
Example of furnished dollhouse.

Children were instructed to furnish a wooden dollhouse (Nic Spiel + Art GmbH, Laupheim, Germany, see Figure 2) with 61 local objects. The dollhouse featured two floors and four rooms, each sized at 31 cm by 40 cm. The standardized initial setup of the dollhouse comprised the following rooms and their characteristic anchor objects: a bedroom with a bed, a kitchen with a stove, a bathroom with a shower, and a living room with a sofa. Each participant was asked to arrange all objects at the place in the house that would be most fitting. There was no time limit. Children’s hands were video-recorded during the furnishing.

#### 2.4.1. Stimuli

Out of 61 dollhouse objects, we chose objects that were characterized as informative based on common sense, as applied in a previous study [51], and based on statistics of object occurrence in real-world image datasets [52], following the approach used by Öhlschläger and Võ with younger children and adults [7]. Essentially, our inclusion of objects in the final analysis depended on their likelihood of strongly associating a scene with a specific category (e.g., a scene that has toilet paper in it strongly associates that scene with a bathroom scene). The expectation was that the information provided by the objects and the scene would complement and reinforce each other. Based on these considerations, in the final analysis, the following 41 objects were included: armchair, baby bed, baby chair, backpack, bath rug, bed, blanket, bread, book, butter, chair, cheese, closet, coffee table, cup, desk, dinner set, dining table, fridge, iron, iron board, jam jar, jug, ketchup, nightstand, pillow, plant, pot, shelf, shower, sink, soap, sofa, stove, trashcan, toilet, toilet paper holder, toilet rug, towel, toothbrush, and vacuum.

#### 2.4.2. Analysis

Semantic knowledge in the dollhouse task was measured as the accuracy of placing objects in their respective rooms, i.e., the number of correctly placed objects (in %). Some objects were semantically consistent for more than one room (e.g., pillow) and counted as correct in any of the respective rooms (e.g., bedroom and living room). Also, some of the objects were not easily recognized by the children (e.g., nightstand) and represented as other similar objects by the children (e.g., stool). We counted these placements as correct.

The measure of syntactic knowledge in the dollhouse task was determined by the distance between the predefined anchor and local objects, given in meters (m) in a 3D environment. To obtain this information, we conducted a 3D scan of each child’s completed dollhouse. These scans were imported into Unity (Unity Technologies, 2023, Version 2021.3.18f1), where we had pre-scanned dollhouse objects. We placed the virtual objects into the scanned dollhouse environment just as the participant placed them in, ensuring precision in our measurements. After positioning all the objects, a script we wrote in Unity generated a matrix showing the distance between the center of every object. Our focus was solely on objects placed in the correct room category, and specifically on the predefined anchor and local objects.

### 2.5. Statistical Analysis

To perform statistical analysis, we utilized linear mixed models (LMMs) [53] and generalized linear mixed models (GLMMs). LMMs allowed us to incorporate the saliency ranks of scenes and participants as random factors. GLMMs, which extend LMMs, were employed to handle data with non-normal distributions or binomial responses, enabling us to use raw data without aggregation [53]. Specifically, GLMMs were used for models with binomial responses, such as object placement performance in the dollhouse task, and when scaling methods could not normalize the response variable distribution. The models were implemented in an R environment (version 2023.09.1; R Core Team, 2021), using the ‘lmer’ function from the lme4 package [54]. The corresponding *p*-values were calculated using the lmerTest package [55], applying Satterthwaite approximations for the degrees of freedom.

In the models for the free-viewing task, dwell time was the fixed factor, while language groups (DLD-TD), violation type (consistent, semantically inconsistent, and syntactically inconsistent) and age groups (“5.58 years–7.92 years”and“8.92 years–10.83 years”) served as predictors. A log transformation was applied to raw dwell time values (measured in ms) in the linear mixed-effects models (lmer). Subjects were always included as a random factor to account for subject variability. We also set a random intercept and random slope for saliency rank in each scene. This approach allows both the baseline response and the saliency rank effect to vary by scene, providing a more flexible model that can capture scene-specific variations in both baseline levels and predictor effects. Tests from which we derived visual intelligence and attention scores, such as CPM, FEW-3, and star search (SET5–10), were included as covariates based on their contribution to the model and their impact on the response variable.

In the dollhouse task models, where the fixed factor is object placement performance or mean distance between related objects, subjects were always included as random factors. The predictors were language groups and age groups. Additionally, CPM, FEW, and SET-3 scores were again incorporated as covariates based on their contribution to the model and their effect on the response variable. The only exception to this was when examining the relationship between dollhouse task measures and language measures; in this case, aggregated data were used and age was added as a covariate factor.

## 3. Results

### 3.1. Tests of Language Development

T-Tests for unpaired samples confirmed a significantly lower performance of children in the DLD group compared to TD children in all subtests of the SET 5–10, as well as in all four scores of the PDSS subtest picture description (see Table 1).

### 3.2. Nonverbal Cognitive Tests

Descriptive statistics and group comparisons of all three nonverbal cognitive tests are displayed in Table 1. In the attention test star search (subtest 3 of SET 5–10), both groups performed equally well and within the age-appropriate norms (percentile rank ≥ 16). In the CPM, performance of both groups was within the age norm (percentile rank ≥ 16). However, children with DLD recorded significantly lower scores than TD children. In the test of visual perception (FEW-3), both groups showed age-appropriate performance (percentile rank ≥ 16), but children with DLD again scored lower than TD children.

### 3.3. Free-Viewing Task

Descriptive statistics as to dwell time on consistent, as well as semantically and syntactically inconsistent, objects, by group and age, are displayed in Table 2.

**Figure 3:**
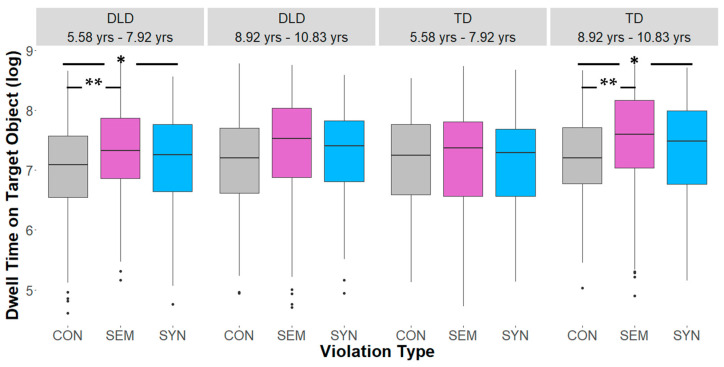
Dwell time on target objects (log, based on raw values in ms) by violation type, group, and age. * indicates significance with *p* < 0.05 and ** indicates significance with *p* < 0.01. CON = Consistent scenes, SEM = Semantically inconsistent scenes, SYN = Syntactically inconsistent scenes.

**Figure 4:**
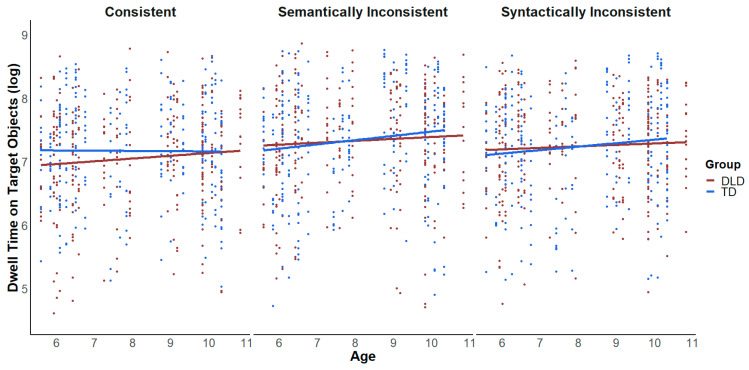
Dwell time on target objects (log, based on raw values in ms) by violation type, as function of age.

Results from the LMER models showed a main effect of violation type on children’s dwell time (*β* = −0.1104, *SE* = 0.02733, *t* = −4.039, *p* < 0.001) with significantly shorter looking times associated with consistent objects, compared to the semantic and syntactic condition (consistency effect). There was no main effect of group on the dwell time of children (*β* = 0.0129, *SE* = 0.0704, *t* = 0.183, *p* = 0.856) and also no main effect of age (*β* = 0.110, *SE* = 0.077, *t* = 1.426, *p* = 0.163). In more detail, both groups showed a semantic inconsistency effect, i.e., they spent less time dwelling on consistent objects compared to semantically inconsistent objects (DLD: *β* = −0.2764, *SE* = 0.0663, *t* = −4.166, *p* < 0.0001; TD: *β* = −0.1683, *SE* = 0.0671, *t* = −2.508, *p* < 0.0328). Children with DLD dwelled longer on the syntactically inconsistent objects compared to consistent objects (*β* = −0.174, *SE* = 0.066, *t* = −2.629, *p* = 0.023). Furthermore, this consistency effect was modulated by the factors of age and group (see Figure 3 and Figure 4): while TD children of the younger age group showed no differences in their dwell time with respect to the three different scene conditions, older children with TD demonstrated a robust consistency effect, i.e., shorter looking times were associated with consistent objects compared to both semantically inconsistent objects (*β* = −0.284, *SE* = 0.097, *t* = −2.930, *p* = 0.0096) and syntactically inconsistent objects (*β* = −0.268, *SE* = 0.094, *t* = −2.844, *p* = 0.0125). Children in the DLD group showed the opposite pattern compared to younger children, displaying a semantic and syntactic inconsistency effect (*β* = −0.3056, *SE* = 0.096, *t* = −3.179, *p* = 0.0043; *β* = −0.2182, *SE* = 0.091, *t* = −2.386, *p* = 0.0452, respectively), while older children with DLD dwelled equally long on consistent and inconsistent objects.

#### Language Skills and Implicit Measure of Scene Knowledge

**Figure 5:**
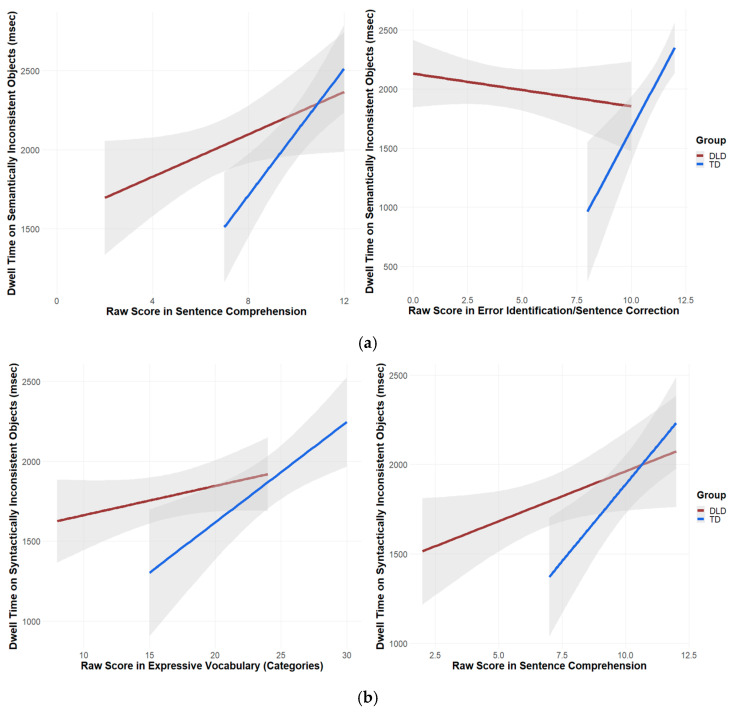
Relationship between language skills and dwell time: (**a**) semantic scene violations; (**b**) syntactic scene violations.

Results from the models revealed significant relationships between language skills and the sensitivity towards semantic and syntactic scene violations (see Figure 5). Children with TD looked longer at the semantically inconsistent objects in cases in which their raw scores on the subtests of sentence comprehension (*β* = 0.107, *SE* = 0.042, *t* = 2.548, *p* = 0.022) and error identification/sentence correction (*β* = 0.191, *SE* = 0.0788, *t* = 2.425, *p* = 0.028) were increased. Children with DLD only showed this relationship for the subtest on sentence comprehension (*β* = 0.040, *SE* = 0.0173, *t* = 2.339, *p* = 0.019). Both groups looked longer at the syntactically inconsistent objects when their raw scores on expressive vocabulary for categories (TD = *β* = 0.054, *SE* = 0.021, *t* = 2.603, *p* = 0.019; DLD = *β* = 0.026, *SE* = 0.008, *t* = 3.366, *p* < 0.001) and their scores on sentence comprehension (TD = *β* = 0.131, *SE* = 0.039, *t* = 3.359, *p* = 0.004; DLD = *β* = 0.049, *SE* = 0.016, *t* = 2.980, *p* = 0.003) increased.

### 3.4. Dollhouse Task

**Figure 6:**
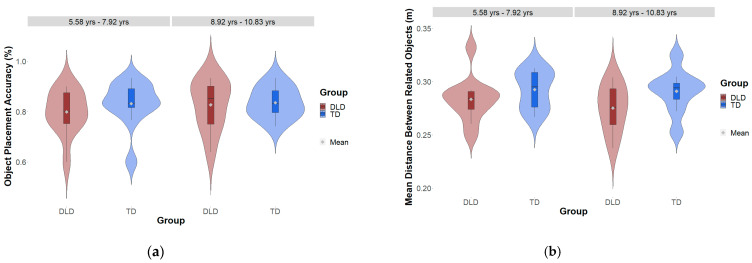
Performance in the dollhouse task by group and age: (**a**) object placement accuracy (%); (**b**) mean distance between related objects (m).

Descriptive statistics relating to object placement accuracy (% of correctly placed objects) and relating to the distance between related objects by group and age are displayed in Table 3 and Figure 6. Results from the GLMM showed no main effect of group (*β* = −0.169, *SE* = 0.151, z = −1.124, *p* = 0.261) or age on the dollhouse semantics, i.e., object placement performance (*β* = −0.294, *SE* = 0.162, z = −0.187, *p* = 0.069), and no interaction effect between group and age (*β* = −0.086, *SE* = 0.255, z = −0.338, *p* = 0.735): there was no difference in the odds ratio between children with and without DLD in either the younger age group (odds Ratio = 1.13, *SE* = 0.225, z- ratio = 0.638, *p* = 0.523) or the older age group (odds Ratio = 1.24, *SE* = 0.245, z- ratio = 1.078, *p* = 0.281). Furthermore, no difference emerged between age groups within the children with DLD (odds Ratio = 1.29, *SE* = 0.272, z- ratio = 1.185, *p* = 0.236). Within the group of TD children, younger children showed a numeric trend towards a better performance (odds Ratio = 1.40, *SE* = 0.282, z- ratio = 1.679, *p* = 0.093).

With respect to dollhouse syntax, i.e., the distance between related objects, with smaller distances indicating higher knowledge about spatial arrangements, the LMM showed no main effect relating to group (*β* = 0.011, *SE* = 0.007, *t* = 1.498, *p* = 0.143) or age (*β* = −0.007, *SE* = 0.008, *t* = −0.922, *p* = 0.363), as well as no interaction effect between group and age (*β* = 0.007, *SE* = 0.013, *t* = 0.570, *p* = 0.572). Children with and without DLD did not show any difference in dollhouse syntax performance within the younger (*β* = −0.007, *SE* = 0.009, *t* = −0.733, *p* = 0.468) and older age groups (*β* = −0.014, *SE* = 0.009, *t* = −1.4703, *p* = 0.151), and the age groups did not show any differences within the group of children with DLD (*β* = 0.109, *SE* = 0.010, *t* = 1.033, *p* = 0.308) and TD (*β* = 0.003, *SE* = 0.009, *t* = 0.353, *p* = 0.726).

Language Skills and Explicit Measure of Scene Knowledge

**Figure 7:**
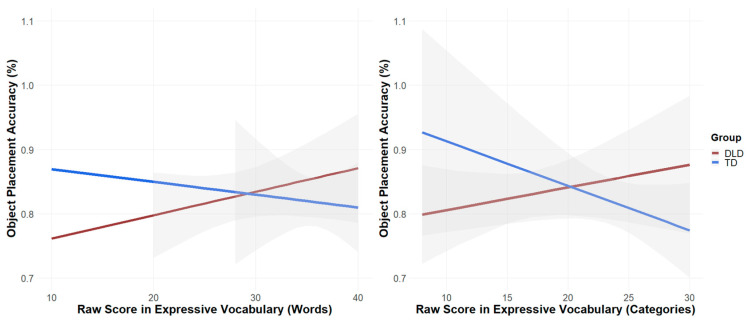
Relationship between expressive vocabulary (words and categories) and accuracy of object placement (%), with CPM score as covariate.

The model’s results show that the language skills of children with DLD predicted their performance in dollhouse semantics, with the % of correct object placement increasing with the subjects’ raw scores as to expressive vocabulary (for categories: *β* = 0.009, *SE* = 0.004, *t* = 2.193, *p* = 0.042 and a numeric trend for words: *β* = 0.006, *SE* = 0.003, *t* = 1.802, *p* = 0.089). Performance in the nonverbal intelligence assessment (CPM-Score) also predicted object placement (*β* = 0.013, *SE* = 0.004, *t* = 3.252, *p* = 0.005). When including CPM scores as a covariate in our model (Figure 7), the effects of expressive vocabulary relative to categories (*β* = 0.005, *SE* = 0.004, *t* = 1.234, *p* = 0.234) and words on children’s object placement performance were no longer significant (*β* = 0.002, *SE* = 0.003, *t* = 0.656, *p* = 0.52). For TD children, including CPM as a covariate in the model revealed an unexpected pattern: object placement performance decreased significantly with increased expressive vocabulary for categories (*β* = −0.014483, *SE* = 0.005, *t* = −2.643, *p* = 0.018).

**Figure 8:**
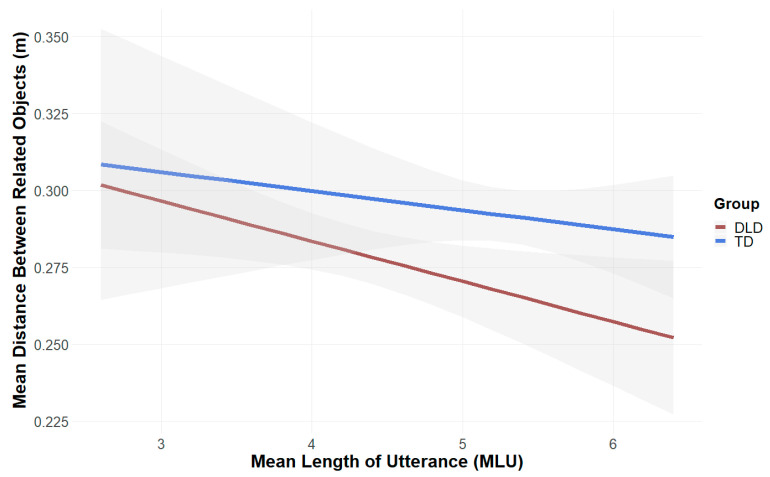
Relationship between mean length of utterance (MLU) and distance of related objects (m, measured in Unity) in the dollhouse task.

Children with DLD showed a trend towards demonstrating a positive influence of MLU (mean number of words per utterance) on their syntactic performance, i.e., the distance between related objects (*β* = −0.012, *SE* = 0.006, *t* = −1.910, *p* = 0.073): children with high MLU scores placed related objects more closely in the dollhouse (see Figure 8). We ran our models for dollhouse syntax and language scores without adding CPM as a covariate, as, in both groups, CPM score did not affect the performance of children (DLD = *β* = 0.00008, *SE* = 0.001, *t* = 0.065, *p* = 0.949; TD = *β* = 0.0009, *SE* = 0.0006, *t* = 1.432, *p* = 0.170) and ANOVA analysis within the models did not suggest a difference.

## 4. Discussion

The aim of the study was to uncover potential differences between primary school-aged children with and without DLD in their efficiency of scene-grammar processing. To this end, we (1) used an implicit task to investigate the processing of semantic and syntactic violations of scene grammar in everyday scenes by analyzing gaze behavior, and (2) asked children to furnish a dollhouse in order to assess their explicit scene-grammar knowledge, i.e., knowing *what* objects (semantics) belong *where* (syntax). The results can be summarized as follows: Overall, children with and without DLD did not differ in their absolute dwell time on consistent targets and neither for semantically nor syntactically inconsistent objects in the free-viewing task. However, inconsistency effects were modulated by the factors of age and group. Older children with TD showed semantic and syntactic inconsistency effects, whereas younger participants did not. In children with DLD, the younger age group showed both inconsistency effects, whereas the older children did not. In the dollhouse task, no group differences were found regarding the accuracy of object placement (semantic scene knowledge) or the distance between related objects (syntactic scene knowledge). In both tasks, high language scores (expressive vocabulary and grammar scores) for children with and without DLD were associated with high performance in scene-grammar processing. In addition, children with DLD showed age-appropriate but significantly poorer performance on the nonverbal cognitive test (CPM) and the visual perception test (FEW-3), compared to the control group.

### 4.1. Group Differences and Communalities in Gaze Behavior Towards Scene Inconsistencies

The results of the implicit measure of scene-grammar processing partially met our expectations. Against the hypothesis, no group differences in absolute dwell times on targets in the three different conditions appeared, meaning that children with and without DLD looked equally long at consistent and at semantically and syntactically inconsistent targets. Dwell times on consistent targets were lowest in all subgroups (younger and older children with DLD and TD), indicating that all children, regardless of age and language ability, implicitly recognized these violations. Some of the children in both groups also verbalized their recognition during or after the task by saying that some objects did not belong at the locations where they were shown. In line with previous research [7,42], semantic inconsistency effects were more pronounced than syntactic inconsistency effects in children with and without DLD, suggesting a developmental trajectory of being able to recognize the more obvious semantic violations at earlier ages, whereas sensitivity to syntactic scene violations develops later. These results indicate a high degree of comparability in gaze behavior towards everyday scenes in children with and without DLD.

However, when age is considered, a different picture is revealed with regard to the emergence of inconsistency effects. As mentioned before, inconsistency effects become stronger with increasing age. The children with TD showed this developmental trajectory, with inconsistency effects for semantic and syntactic violations being absent in the earlier age group and present in the older one. In the DLD group, only the younger children showed inconsistency effects. This finding contrasts with previously reported developmental trajectories [7] and may be due to the following reason: the difference in dwell time was largest between the two young age groups, with and without DLD, for the consistent condition. In accordance with the developmental trajectory, the dwell time associated with consistent objects remained constant or decreased and dwell times associated with the semantic and syntactic condition increased as the TD children become older. However, dwell times of children with DLD increased in all three conditions which is why the greatest increase was observed for the consistent condition. In line with the procedural deficit hypothesis (PDH), this pattern may reflect compensatory strategies, indicating a greater need for prolonged exploration of typical (consistent) scenes in order to extract scene regularities before violations within scenes can become the focus of attention. Thus, children with DLD seemed to show a different or delayed developmental trajectory relative to the control group, one characterized by a prolonged preference for looking at consistent rather than inconsistent scenes. The difference in dwell times between conditions decreases in older children, because dwell times for consistent objects still increase, and to a greater extent than dwell times for semantically and syntactically inconsistent objects. As a result, the apparent inconsistency effect disappears. Thus, children with DLD seem to focus persistently on consistent objects, rather than showing a growing interest in scene violations. The absence of stable inconsistency effects in children with DLD confirms the results of Helo and colleagues [42], who found inconsistency effects in this group only for the mixed condition (semantic plus syntactic violation), but no effects for either semantic-only or syntactic-only violations. Our inclusion of older children of primary-school age revealed a delay in the development of scene grammar in relation to DLD, which (1) persists beyond preschool age and (2) is more pronounced in the perception of syntactic scene inconsistencies.

As expected, we found stable relationships between language skills and the efficiency of scene-grammar processing, confirming previous research [7,42]. Both groups showed a higher sensitivity to semantic and syntactic scene violations (expressed in longer dwell times) the higher their scores were in expressive vocabulary and grammar skills (sentence comprehension, error identification, and MLU). Thus, language and visuospatial processing appear to be linked more closely than is often assumed. Such a link could also explain the lower performance of the children with DLD in the visual perception test, again supporting a domain-general view on DLD. According to this account, processing deficits in superordinate higher cognitive functions, such as the central executive being part of WM, which integrates visual and linguistic information, contribute to the manifestation of DLD. Taken together, the development of sensitivity towards scene violations in children with DLD appears to lag behind due to the influence of language skills on scene-grammar processing.

### 4.2. Group Differences and Communalities in Object Placement Accuracy and Object Distance in the Dollhouse Task

Against our hypothesis, the two groups did not differ in their accuracy of object placement and in the distance between the arranged local objects and their anchors. Children with and without DLD placed objects in the appropriate rooms with a very high mean accuracy of 82%. In addition, there was no difference between the younger and older children. Combined with a similar study by Öhlschläger and Võ [7], who applied the dollhouse task with TD children aged two to four years, our results suggest an age-related progression of explicit scene knowledge, from 25% correctly placed objects in 2-year-olds to 82% in 6–11-year-olds. The lack of group effects may be due to the relatively low demands of this task for primary school-aged children and the fact that most of the objects were highly frequent and familiar. In future studies, it would be interesting to investigate whether there are group differences when the task is performed with low-frequency objects and more rooms, such as a home office, children’s room, or garage.

In line with your hypothesis, the participants’ language skills predicted their performance in object placement and distance of objects. However, this relationship was also influenced by the factors of group and nonverbal cognitive ability (CPM score): only children with DLD showed higher performance in object placement in cases in which their vocabulary score was higher. Since this correlation was only present in children with DLD and disappeared when controlling for the CPM score, this result must be interpreted with caution.

One explanation for the influence of language ability on performance in the dollhouse task could be that the children solved this seemingly nonverbal task verbally, using inner speech and self-talk, e.g., about object names, properties or appropriate rooms, as they placed the objects. Children with poorer language skills placed objects more often incorrectly and at greater distances from their anchors. Poorer language skills when attempting to solve this task verbally may have had a negative effect on task performance, or, at least, self-talk was not as supportive as for children with TD. Language ability may also have played a role in the lower performance of the DLD group in the visual perception test, as the children often solved the task by thinking aloud.

### 4.3. Limitations and Future Research

The findings of the present study are based on rather small sample sizes, especially for the age-relative group comparison. This should be taken into consideration when interpreting the results, as the results may be less generalizable. In addition, the heterogeneity of the groups may have further influenced the results as children diagnosed with DLD tend to show very different linguistic profiles (e.g., focus on lexical semantic or grammatical problems). Another factor may be the different degrees of severity of DLD. The sample of children with DLD in this study consisted of children affected rather severely by the condition. Conversely, this means that the results cannot be simply extrapolated to individuals with DLD who are less severely affected. To substantiate the findings regarding the efficacy of scene-grammar processing in the context of DLD, it is thus imperative to confirm the results with a larger sample size, with subgroups of children showing lexical–semantic or grammatical deficits only, and with a second control group of younger TD children with similar language levels compared to the clinical group.

Furthermore, the exact influence of nonverbal cognitive abilities on scene processing, as tested by the CPM, remains an open question. The relationship was significant only in children with DLD, who also showed reduced performance on the visual perception test FEW-3. The CPM can be considered a visual form-related task, comparable to the FEW-3. Therefore, it cannot be completely ruled out that the apparent correlation between nonverbal cognitive performance and visuospatial scene processing is an artifact of the similarity between the two tasks. Future studies should therefore apply a wider range of tests for nonverbal intelligence with different cognitive demands.

In addition to the dollhouse task, which could be modified in terms of complexity, explicit knowledge of scene grammar could also be evaluated using other behavioral tasks. For instance, participants could be asked to correct scene violations that have been presented to them, to describe prototypes of scenes, or to identify and add missing local and anchor objects.

In subsequent research, it would be worthwhile to concentrate on cross-linguistic comparisons of scene-grammar development and to consider cultural differences. This is because children are raised in different environments that are shaped by their culture. This also affects how the same indoor scenes are processed in different cultural contexts. It would therefore be interesting to investigate how the perception of scene-grammar develops in children growing up across various cultures.

## 5. Conclusions

Overall, the study suggests subtle impediments in visuospatial processing in children with DLD, involving not only the perception and processing of abstract visual forms and patterns, but also relating to real-world visual scenes that constitute the external environment and thus provide a framework for language acquisition. Supporting the domain-general view of DLD, this finding also has clinical relevance. For instance, the existence of unperceived problems in components of nonverbal WM (central executive or visuospatial sketchpad) which manifest themselves in altered visuospatial processing has the potential to result in delays in the progress of therapy, particularly if visual materials are utilized extensively during treatment. Furthermore, the acquisition of mental concepts pertaining to spatial-positional relationships, including linguistic knowledge such as word meanings, word forms, and grammar rules, may be influenced by visuo-spatial perception that differs from that of typically developing children. Thus, it may be worthwhile to consider children’s nonverbal WM capacities in the diagnostic process in order to more accurately determine the specific intervention needs of each individual child with DLD.

## Figures and Tables

**Table 1:** Descriptive statistics and group statistics for the verbal and nonverbal developmental tests.

	Subtests	PRs ^1^ DLD *M* (SD)	PRs TD *M* (SD)	t-Tests for Unpaired Samples
Verbal Tests	SET 5–10:			
	Expressive Vocabulary (Words)	19.6 (19.7)	73.5 (24.9)	*t*(38) = −7.60, *p* = <0.001
	Expressive Vocabulary (Categories)	28.5 (23.4)	79.8 (23.9)	*t*(38) = −6.85, *p* = <0.001
	Sentence Comprehension	25.3 (27.2)	58.0 (32.6)	*t*(38) = −3.44, *p* = 0.001
	Text Comprehension	33.8 (34.0)	79.5 (29.0)	*t*(38) = −4.58, *p* = <0.001
	Error Identification/Sentence Correction	7.45 (11.5)	75.1 (27.9)	*t*(38) = −10.02, *p* = <0.001
	PDSS—In-Depth Grammar Analysis (Picture Description Task):			
	MLU	29.5 (25.8)	69.3 (23.4)	*t*(38) = −5.10, *p* = <0.001
	Completeness	10.0 (12.1)	51.7 (31.9)	*t*(38) = −8.09, *p* = <0.001
	Score A (use of target structures)	36.3 (28.4)	70.0 (14.4)	*t*(38) = −4.73, *p* = <0.001
	Score B (use of non-target structures)	12.5 (23.1)	70.2 (30.5)	*t*(38) = −6.75, *p* = <0.001
Nonverbal Tests	SET 5–10:			
	Attention Test (Star Search)	26.6 (26.8)	35.9 (22.1)	*t*(38) = −1.19, *p* = 0.241
	CPM	35.6 (28.6)	60.0 (23.3)	*t*(38) = −2.95, *p* = 0.005
	FEW-3			
	Figure-Ground	29.3 (32.1)	49.3 (28.0)	*t*(38) = −2.10, *p* = 0.042
	Visual Closure	33.3 (26.8)	54.2 (7.5)	*t*(38) = −2.43, *p* = 0.020
	Form Constancy	20.1 (51.9)	51.5 (29.5)	*t*(38) = −3.83, *p* = <0.001

^1^ PR = Percentile rank.

**Table 2:** Descriptive statistics for dwell time in ms by violation, group, and age.

Group	Age (Years)	Violation	Mean (*M*)	Standard Error (*SE*)	Minimum	Maximum
DLD	5.58–7.92	Consistent	1439	90	100	5762
DLD	8.92–10.83	Consistent	1649	102	140	6512
DLD	5.58–7.92	Semantic	1939	125	174	7068
DLD	8.92–10.83	Semantic	2092	114	110	6346
DLD	5.58–7.92	Syntactic	1741	101	116	5194
DLD	8.92–10.83	Syntactic	1826	97	140	5370
TD	5.58–7.92	Consistent	1691	100	168	5090
TD	8.92–10.83	Consistent	1690	107	152	5784
TD	5.58–7.92	Semantic	1825	112	112	6234
TD	8.92–10.83	Semantic	2390	139	134	6364
TD	5.58–7.92	Syntactic	1631	100	170	5846
TD	8.92–10.83	Syntactic	2147	136	172	6058

**Table 3:** Descriptive Statistics as to object placement and distance between related objects in the dollhouse task.

Measure	Group	Age (Years)	Mean (*M*)	Standard Error (*SE*)	Minimum	Maximum
Object placement accuracy (%)	DLD	5.58–7.92	79	3	60	90
	DLD	8.92–10.83	83	3	64	93
	TD	5.58–7.92	83	3	60	93
	TD	8.92–10.83	84	2	74	93
Distance between related objects in m (measured in Unity)	DLD	5.58–7.92	0.28	0.01	0.25	0.33
	DLD	8.92–10.83	0.28	0.24	0.24	0.30
	TD	5.58–7.92	0.29	0.27	0.27	0.31
	TD	8.92–10.83	0.29	0.25	0.25	0.33

## Data Availability

Link to the SCEGRAM Database [4]: https://www.scenegrammarlab.com/databases/scegram-database/, accessed on 23 January 2025.
